# Analysis of PET parameters as prognosticators of survival and tumor extent in Oropharyngeal Cancer treated with surgery and postoperative radiotherapy

**DOI:** 10.1186/s12885-021-08035-9

**Published:** 2021-03-25

**Authors:** Kyu Hye Choi, Jin Ho Song, Eun Young Park, Ji Hyun Hong, Ie Ryung Yoo, Youn Soo Lee, Dong-Il Sun, Min-Sik Kim, Yeon-Sil Kim

**Affiliations:** 1 Department of Radiation Oncology, Seoul St. Mary’s Hospital, College of Medicine, The Catholic University of Korea, Seoul, Republic of Korea; 2 Department of Nuclear Medicine, Seoul St. Mary’s Hospital, College of Medicine, The Catholic University of Korea, Seoul, Republic of Korea; 3 Department of Pathology, Seoul St. Mary’s Hospital, College of Medicine, The Catholic University of Korea, Seoul, Republic of Korea; 4 Department of Otorhinolaryngology, Seoul St. Mary’s Hospital, College of Medicine, The Catholic University of Korea, Seoul, Republic of Korea

**Keywords:** Oropharyngeal cancer, PET, SUVmax, Depth of invasion, Extranodal extension

## Abstract

**Background:**

Positron-emission tomography (PET) is widely used to detect malignancies, but consensus on its prognostic value in oropharyngeal cancer has not been established. The purpose of this study was to analyze the PET parameters associated with tumor extent and survival in resectable oropharyngeal cancer.

**Methods:**

The PET parameters in oropharyngeal cancer patients with regional node metastasis who underwent surgery and postoperative radiotherapy between January 2005 and January 2019 were analyzed. We calculated the SUVmax, tumor-to-liver ratio (TLR), metabolic tumor volume (MTV, volume over SUV 2.5), and total lesion glycolysis (TLG, MTV x mean SUV) of the primary lesion and metastatic nodes. Histologic findings, patient survival, and recurrence were reviewed in the medical records.

**Results:**

Fifty patients were included, and the PET parameters were extracted for 50 primary lesions and 104 nodal lesions. In the survival analysis, MTV and TLG of the primary lesions showed significant differences in overall survival (OS) and recurrence-free survival (RFS). In the multiple regression analysis, TLG of the primary lesion was associated with the depth of invasion (DOI). MTV of the nodes was a significant factor affecting extranodal extension (ENE).

**Conclusions:**

PET parameters could be related with OS, RFS, DOI of the primary tumor, and ENE. PET would be expected to be a useful diagnostic tool as a prognosticator of survival and pathologic findings in oropharyngeal cancer.

## Background

With the development of functional imaging, positron-emission tomography (PET) has been used as a diagnostic method by tracking metabolic activity with radioactive isotopes that emit positrons [1].The most commonly used ^18^F-fluorodeoxyglucose (^18^F-FDG) radioactive isotope is a glucose-like substance, which shows active glucose metabolism in lesions such as cancer, and it can be a useful tool for detecting cancer spread in the body. By combining PET-scanning with computed tomography (CT), anatomical information and accurate image correction can be achieved [2].

PET provides objective and reproducible information through standardized uptake values (SUV), from which a quantitative factor is extracted, and the maximum SUV is known to be related to survival rate in several solid tumors [3–5]. Previous studies on the association between PET parameters and prognosis have been actively conducted, but consensus has not established the application of PET as a staging tool or its prognostic value in solid tumors [6–8]. PET is usually used as supplementary anatomic imaging for assessing tumors or nodal extension in clinical settings.

In head and neck cancer, the nodal stage is based on several studies in which the extranodal extension (ENE) of the lymph nodes (LNs) affected the prognosis [9–11]. It is also known that the depth of invasion (DOI) of the primary tumors is an important prognostic factor associated with local recurrence [12, 13]. The expression of human papillomavirus (HPV) has recently been studied as a factor affecting the prognosis of patients with oropharyngeal cancer, and different staging systems have been established according to HPV status [14, 15]. The purpose of this study was to analyze the association between surgical histologic findings of oropharyngeal cancer and PET parameters before treatment and assess their prognostic role in survival and pathology.

## Methods

### Patient population

This was a retrospective study conducted on patients who were diagnosed with resectable oropharyngeal cancer and underwent surgery from January 2005 to January 2019. The resectability was based on the technical ability to clear margins and the possibility of total cancer removal in primary site and involved LNs. The eligibility criteria were: 1) primary tumor stage T1–4 and regional node metastasis without distant metastasis at initial diagnosis; 2) squamous cell carcinoma confirmed through biopsy; 3) PET-CT examination before treatment; 4) patients who underwent curative resection with LN dissection and completed adjuvant chemoradiotherapy or radiotherapy as needed; and 5) patients with Karnofsky scores ≥70 and ages of 19–70 years. Patients who underwent neoadjuvant chemotherapy before surgery were excluded. Primary tumor resection was conducted with wide excision in an effort to obtain enough surgical margin. Modified radical neck dissection was done for the clinically positive cervical nodes, and selective neck dissection was done as an elective neck treatment on the clinically negative neck node region. The indications and modalities for adjuvant (chemo)radiotherapy were cases of positive or close margins found on the resection, multiple lymph-node metastasis, extranodal extension of involved LNs, advanced T classification, lymphovascular invasion, and perineural invasion requiring additional treatment.

This study was conducted in accordance with the Declaration of Helsinki. The Institutional Review Board of the Catholic Medical Center, the Catholic University of Korea approved the study protocol (No. KC19RISI0812). Informed consent was waived due to the retrospective study design by the same ethics committee that approved this study (The Institutional Review Board of the Catholic Medical Center, the Catholic University of Korea).

### Treatment technique and ^18^F-FDG PET-CT protocol

All patients fasted for ≥6 h before the PET-CT scans and were in the supine position during scanning. There were no patients with blood glucose levels above 150 mg/dL before injection. The intravenous injection of 3.7–5.5 MBq/kg of ^18^F-FDG started the scan 60 min later. Intravenous contrast agent was not administered and images were acquired using a combined PET-CT in-line system with a Biograph Duo or Biograph TruePoint (Siemens Medical Solutions, Knoxville, TN, USA) and a Discovery 710 (GE Healthcare, Milwaukee, WI, USA).

### Target volume delineation and imaging analysis

SUV was calculated using a standard calibration for body weight and injection dose. The primary tumor and metastatic nodes were identified in the region-of-interest (ROI). The maximum SUV (SUVmax), metabolic tumor volume (MTV), and total lesion glycolysis (TLG) were extracted from the analysis of the PET parameters. The primary tumor was delineated using CT and the metastatic nodes more than 1 cm in longest diameter with a minimal SUVmax of 1.5 were included in analysis. Correction by mean SUV of liver was used to compute the ratio of the tumor-to-liver SUV (TLR) to compensate for the difference due to the three different scanners [16, 17]. For mean SUV of liver, three non-overlapping spherical volumes of interest (VOIs) with a diameter of 3 cm in the liver (2 in the right lobe and 1 in the left lobe) were drawn and the average value was calculated. The MTV was defined by an SUV volume of ≥2.5 in the ROI and the TLG was calculated as MTV x mean SUV. MIM software version 6.8.3 (MIM Software Inc., Cleveland, OH, USA) was used to extract these parameters, and each volume was determined within the ROI by the review software.

### Statistical analysis

The surgical pathology reports of the patients were reviewed to determine the DOI of the primary tumor, the ENE of metastatic nodes, and other important pathologic findings in oropharyngeal cancer. A receiver operating characteristic (ROC) curve was used to evaluate the prognostic power of volumetric factor for recurrence and Pearson’s correlation coefficients were calculated to evaluate the relationship between PET parameters and the pathologic findings. Overall survival (OS) was defined as the time from the date of curative surgery to death and recurrence-free survival (RFS) was defined as the time from the date of curative surgery until the first evidence of disease recurrence. Subsequent follow-up for death and recurrence were recorded, and the OS and RFS were evaluated using Kaplan-Meier curves and the log-rank test. Univariate and multivariate regression models with logistic regression analyses were performed to analyze the factors associated with surgical pathologic findings. The statistical analyses were conducted using SPSS software version 24 (IBM, Armonk, NY, USA).

## Results

### Patient and PET parameters characteristics

During the study period, 252 patients with oropharyngeal cancer were treated at Seoul St. Mary’s Hospital. Of these, 74 patients underwent PET-CT before treatment and a total of 50 patients, except for 24 patients who underwent neoadjuvant chemotherapy, were included in the study. The PET parameters were extracted for 50 primary lesions and 104 nodal lesions. Among the 104 nodes, 38 were ENE-positive nodes, 47 were negative, and the ENE status was unavailable on 19 nodes. The range of the values in the primary tumors (T) was 4.49–28.7 (median, 9.92) for T-SUVmax, 2.07–13.41 (median, 4.57) for T-TLR, 2.65–64.62 cm^3^ (median, 10.89 cm^3^) for T-MTV, and 8.63–619.51 cm^3^ (median, 40.29 cm^3^) for T-TLG. There was no difference in mean SUV values of liver between the high and low TLR groups. The range of the variables for each of the 104 metastatic nodes (N) was calculated as follows: N-SUVmax, 2.66–19.86 (median, 6.71); N-TLR, 1.10–8.84 (median, 3.07); N-MTV, 0.05–133.87 cm^3^ (median, 2.90 cm^3^); and N-TLG, 0.13–904.41 cm^3^ (median, 10.18 cm^3^). To evaluate the metastatic nodal burden of each patient, the parameters of the total metastatic nodes were further analyzed. For the total metastatic nodes (tN) in the patients, the median tN-SUVmax was 8.13 (range, 2.95–19.86), the median tN-TLR was 3.53 (range, 1.10–8.84), the median tN-MTV was 10.38 cm^3^ (range, 0.25–133.87 cm^3^), and the median tN-TLG was 39.69 cm^3^ (range, 0.72–904.41 cm^3^). The patient, treatment, and tumor characteristics are listed in Table 1.

**Table 1:** Patient, treatment (A), and tumor (B) characteristics (*N* = 50)

Characteristic	N (%) or median (range)
(A)	
Patient characteristics	
Age	59 (29–75)
Primary site	
Tonsil	41 (82)
Base of tongue	7 (14)
Soft palate	2 (4)
Sex	
Male	45 (90)
Female	5 (10)
Smoking history	
Non-smoker	17 (34)
Smoker ≤10 PPY	5 (10)
Smoker > 10 PPY	28 (56)
Treatment characteristics	
Primary surgery	
Wide excision	26 (52)
Transoral robotic surgery	24 (48)
Ipsilateral neck dissection	
Radical neck dissection	4 (8)
Modified radical neck dissection	46 (92)
Contralateral neck dissection	
Modified radical neck dissection	4 (8)
Selective neck dissection	37 (74)
Not-done	9 (18)
Adjuvant treatment	
Chemoradiotherapy	35 (70)
Radiotherapy	15 (30)
**(B)**	
T stage	
T1	24 (48)
T2	24 (48)
T3-T4	2 (4)
Depth of invasion (mm)	9 (7–37)
LN number	
1–4	36 (72)
> 4	14 (28)
Largest LN size (cm)	
≤ 3	26 (52)
> 3 but ≤6	19 (38)
> 6	1 (2)
N/A	4 (8)
Extranodal extension	
Negative	21 (42)
Positive	29 (58)
Tumor grade	
Well to moderately	30 (60)
Poorly	16 (32)
N/A	4 (8)
HPV status	
Negative	18 (36)
Positive	27 (54)
N/A	5 (10)
Surgical margin	
Negative	35 (70)
Positive or close	15 (30)
Lymphatic invasion	
Yes	29 (58)
No	21 (42)
Vascular invasion	
Yes	4 (8)
No	46 (92)
Perineural invasion	
Yes	5 (10)
No	45 (90)

*Abbreviation: PPY* packs per year, *LN* lymph node, *N/A* not-available, *HPV* human papilloma virus

### Volumetric parameters and survival

**Fig. 1:**
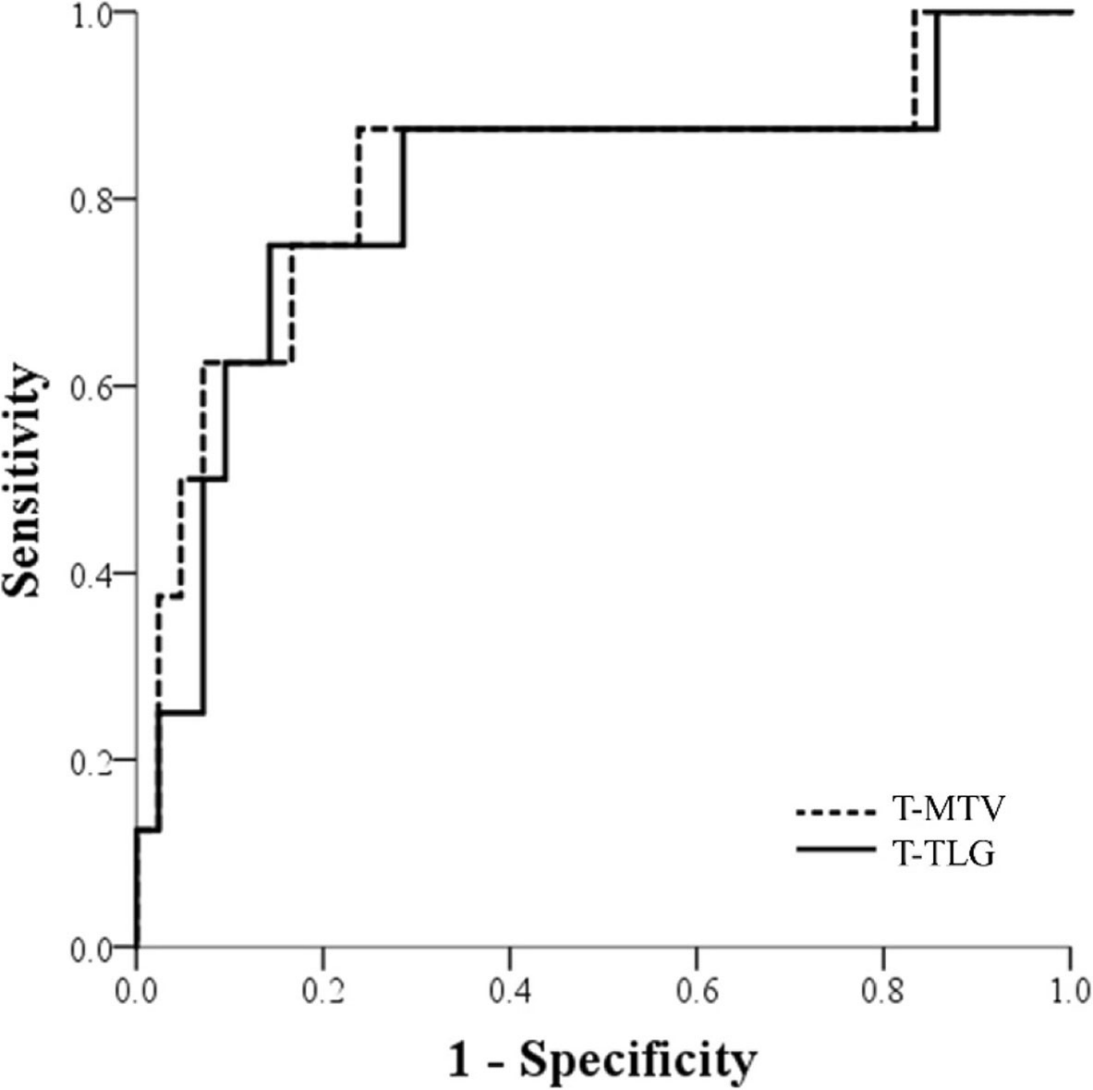
Receiver operating characteristic curve for recurrence in T-MTV and T-TLG. T-MTV = metabolic tumor volume of primary tumor, T-TLG = total lesion glycolysis of primary tumor

**Fig. 2:**
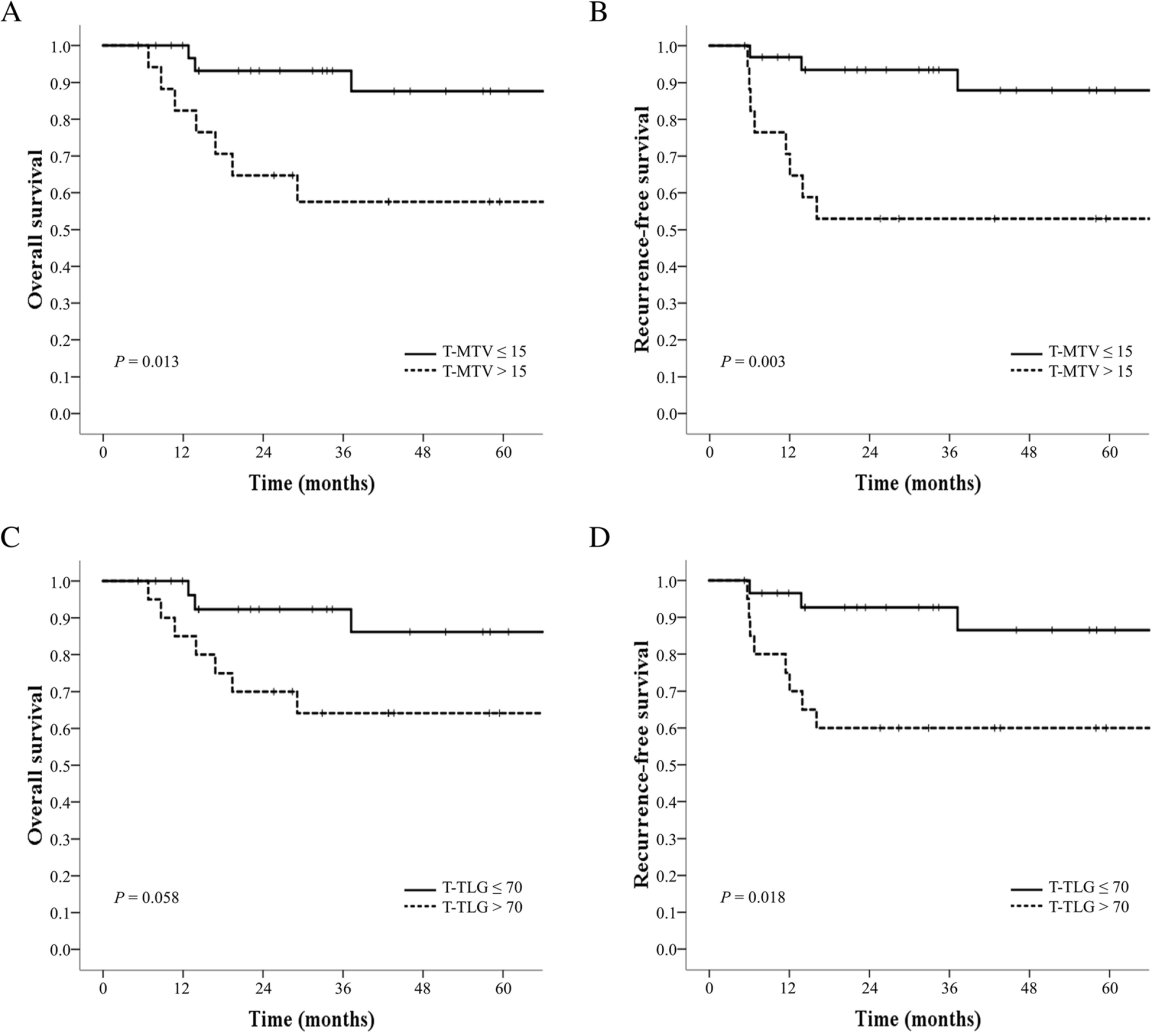
Survival curves of OS and RFS according to T-MTV (**a**, **b**) and T-TLG (**c**, **d**). OS = overall survival, RFS = recurrence-free survival, T-MTV = metabolic tumor volume of primary tumor, T-TLG = total lesion glycolysis of primary tumor

In the median 90.75 months of follow-up, there were eight (16%) recurrences and 12 (24%) deaths. A ROC curve was used to evaluate the prognostic power of T-MTV and T-TLG for recurrence (Fig. 1). The area under the curve (AUC) showed fair prognostic power of 0.824 and 0.807 for T-MTV and T-TLG, respectively (T-MTV: *P* = 0.004, 95% confidence interval (CI): 0.636–1.000; T-TLG: *P* = 0.006, 95% CI: 0.613–1.000). After ROC curve generation, OS and RFS were analyzed by dividing T-MTV 15 cm^3^ and T-TLG 70 cm^3^ by their cutoff values with high sensitivity and specificity. The analysis of OS and RFS for T-MTV showed significant differences between the T-MTV ≤ 15 cm^3^ and > 15 cm^3^ groups (OS, *P* = 0.013; RFS, *P* = 0.003). The survival analysis for T-TLG showed significant differences in RFS (*P* = 0.018) and marginally significant differences in OS (*P* = 0.058) between the T-TLG ≤ 70 cm^3^ and > 70 cm^3^ groups. Figure 2 shows the Kaplan-Meier curves of OS and RFS for T-MTV and T-TLG.

Survival analysis based on metastatic nodes was performed by setting the cutoff values for tN-MTV to 10 cm^3^ and tN-TLG to 35 cm^3^, which showed high sensitivity and specificity in the ROC curves for recurrence. There was no significant difference between tN-MTV and tN-TLG in the log-rank test for OS (*P* = 0.272 in tN-MTV, 0.088 in tN-TLG). Survival analysis for RFS showed no significant difference in tN-MTV (*P* = 0.136), whereas it showed significant difference in tN-TLG (*P* = 0.039).

In summary, the T-MTV and T-TLG of primary tumors were statistically significant prognostic factors for OS and RFS. Among the metabolic parameters of metastatic LNs, tN-TLG also showed prognostic power.

### PET parameters and pathology of the primary tumors

**Fig. 3:**
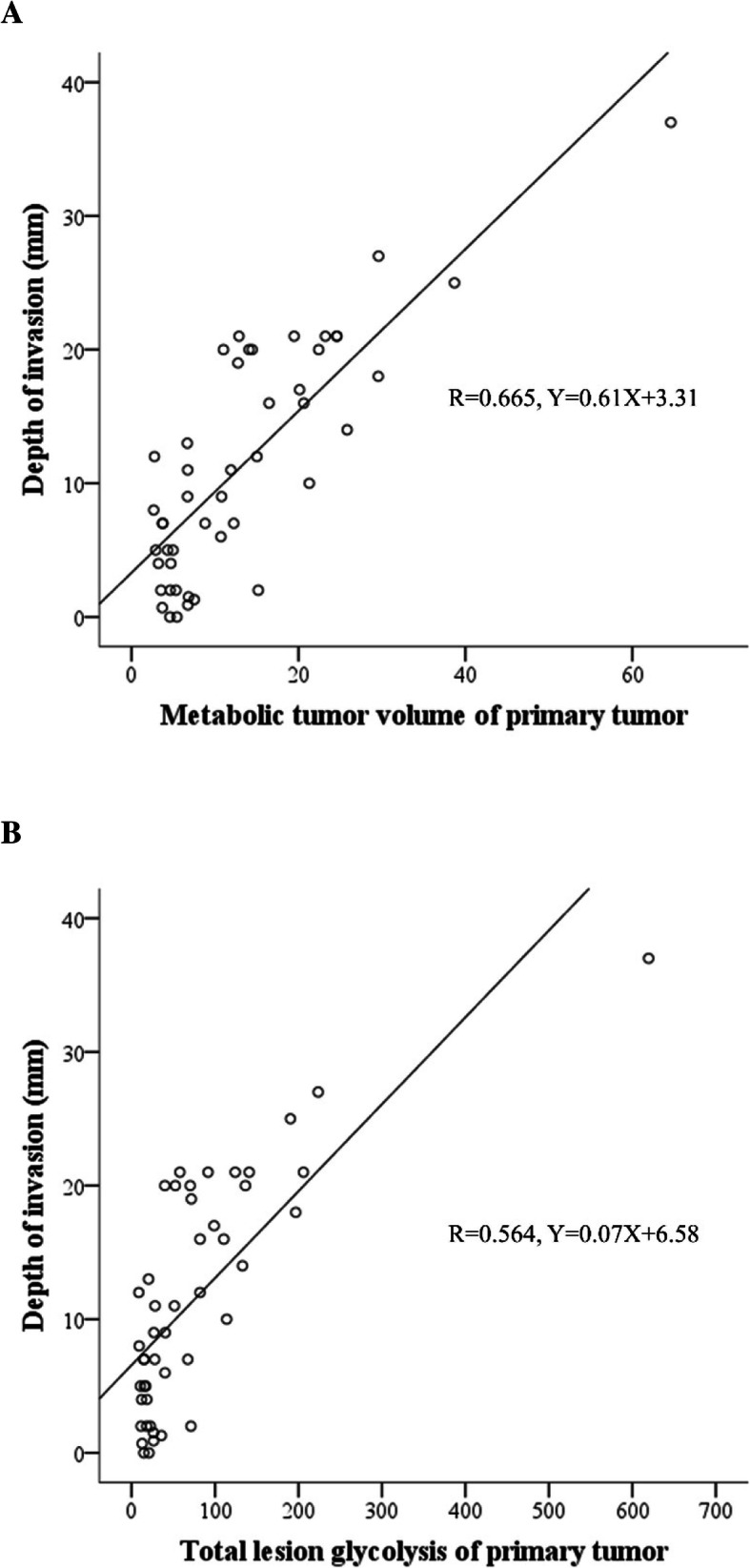
Correlation between the depth of invasion and volume-based parameters (**a**: T-MTV, **b**: T-TLG) of the primary tumors. Volume-based parameters, such as T-MTV and T-TLG in PET-CT as functional images, were useful for T-staging to determine the tumor extent. T-MTV = metabolic tumor volume of primary tumor, T-TLG = total lesion glycolysis of primary tumor

**Fig. 4:**
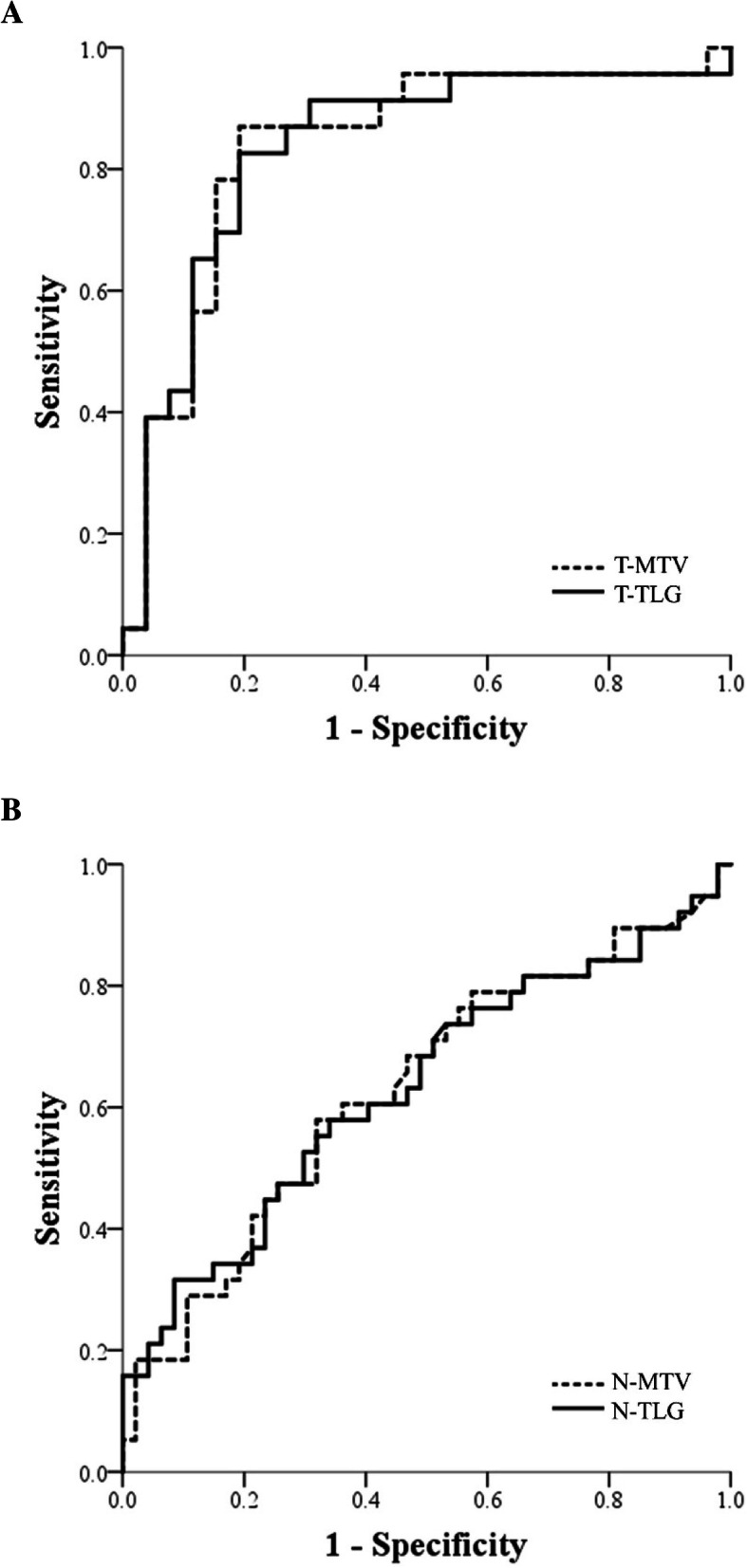
Receiver operating characteristic curve predicting (**a**) depth of invasion (> 10 mm) in the primary tumor for metabolic tumor volume and total lesion glycolysis and (**b**) extranodal extension for nodal total lesion glycolysis. T-MTV = metabolic tumor volume of primary tumor, T-TLG = total lesion glycolysis of primary tumor, N-MTV = metabolic tumor volume of metastatic node, N-TLG = total lesion glycolysis of metastatic node

The relationship between T-MTV and T-TLG and the DOI of the primary tumor is shown in Fig. 3. Pearson’s correlation coefficients were 0.815 for T-MTV and 0.751 for T-TLG (*P* < 0.001, both), representing good correlation. ROC analysis was performed for determining the cutoff points. The AUC of T-MTV and T-TLG were 0.836 (95% CI: 0.712–0.957) and 0.834 (95% CI: 0.715–0.958), respectively, demonstrating fair prognostic value for deep invasive tumors (DOI > 10 mm) (Fig. 4a). T-MTV above 10 cm^3^ and high T-TLG above 45 cm^3^ were cutoff points with high sensitivity and specificity for DOI. Logistic regression (Table 2A) analysis was performed to correlate the DOI with metabolic parameters of the primary tumor and other pathologic factors. T-MTV and T-TLG showed statistically significant differences in univariate analysis for DOI > 10 mm (MTV, *P* = 0.003; TLG, *P* < 0.001). T-TLG was also analyzed in multivariate analysis as a significant associated factor (*P* < 0.001; odds ratio = 13.143; 95% CI: 3.292–52.466). However, neither any other PET parameters, the HPV status, nor histologic variables, such as adjacent structure involvement, lymphovascular or perineural invasion, were associated with the DOI.

**Table 2:** Univariate and multivariate logistic regression analyses of DOI of primary lesion (A) and ENE of metastatic nodes (B)

	UVA	MVA	OR (95% CI)
**(A)**			
T-Max (≤10 vs. > 10)	0.003	0.691	
T-MTV (≤15 cm^3^ vs. > 15 cm^3^)	0.003	0.348	
T-TLG (≤70 cm^3^ vs. > 70 cm^3^)	< 0.001	< 0.001	13.143 (3.292–52.466)
HPV (positive vs. negative)	0.627		
Ki-67 (≤70% vs. > 70%)	0.197		
Lymphovascular invasion (yes vs. no)	0.206		
Perineural invasion (yes vs. no)	0.999		
Tumor grade (well vs. moderate vs. poor)	0.405		
(B)			
N-Max (≤11 vs. > 11)	0.021	0.132	
N-MTV (≤4 cm^3^ vs. > 4 cm^3^)	0.018	0.016	2.933 (1.205–7.138)
N-TLG (≤15 cm^3^ vs. > 15 cm^3^)	0.330		
HPV (positive vs. negative)	0.081		
Ki-67 (≤70% vs. > 70%)	0.229		
Lymphovascular invasion (yes vs. no)	0.330		

*Abbreviation: DOI* depth of invasion, *ENE* extranodal extension, *T-Max* maximum standardized uptake value of primary tumor, *T-MTV* metabolic tumor volume of T, *T-TLG* total lesion glycolysis of T, *HPV* human papillomavirus, *UVA* univariate analysis, *MVA* multivariate analysis, *OR* odds ratio, *CI* confidence interval, *N-Max* maximum standardized uptake value of metastatic node, *N-MTV* metabolic tumor volume of metastatic node, *N-TLG* total lesion glycolysis of metastatic node

### PET parameters and extranodal extension

The PET parameters were analyzed for 85 nodes to determine whether they matched well-known pathological variables of ENE. ROC curves were generated in the analysis of the prognostic value of N-MTV and N-TLG for ENE. The AUCs were 0.630 (95% CI: 0.509–0.751) and 0.631 (95% CI: 0.510–0.753), respectively, (Fig. 4b) and cutoff values of 4 cm^3^ for tN-MTV and 15 cm^3^ for tN-TLG were obtained (N-MTV: sensitivity = 59%, specificity = 67%; N-TLG: sensitivity = 54%, specificity = 64%). In the evaluation of ENE with PET parameters and pathologic variables, the univariate logistic regression analysis showed that N-SUVmax (*P* = 0.021) and N-MTV (*P* = 0.018) were statistically significantly associated with ENE. And among them, N-MTV was significantly different in multivariate analysis for ENE (*P* = 0.016; odds ratio = 2.933; 95% CI: 1.205–7.138). The logistic regression analyses of ENE are summarized in Table 2B. In summary, N-MTV could be a useful parameter for the pathologic invasiveness of metastatic nodes.

## Discussion

In head and neck cancer, evaluating the patient’s prognosis and disease extent through PET images before surgery can reduce complications related to surgery and increase the cure rate [18]. Prior to this, some studies on the prognostic value of PET parameters have been reported [19, 20]. Our data have several meaningful perspectives, including the study design that analyzed PET variables in the preoperative staging process for resectable oropharyngeal cancer, and additionally evaluated the association with surgical pathologic findings.

In previous retrospective studies of SUV factors as prognosticators of head and neck cancer patients, the MTV or TLG of the primary tumors and metastatic lymph nodes were determined to be strong prognostic factors [20–23]. Most of them included various subsites of the head and neck and patients treated with induction chemotherapy or definitive chemoradiation therapy in locally advanced-stage cancer. We particularly analyzed PET parameters associated with surgical pathology and survival in patients with oropharyngeal cancer who underwent surgery without neoadjuvant treatment. Since this study was designed for patients undergoing surgery and postoperative radiotherapy, it was possible to simultaneously confirm the clinical results after surgery and radiotherapy, and analyze the ability of pathologic parameters to related with OS and RFS using PET parameters in these homogeneously treated patients. In addition, by excluding patients who were treated with neoadjuvant treatment, variables related to neoadjuvant treatment could be excluded in analyzing the association of pathological findings with PET before surgery.

In this study, SUVmax, MTV, and TLG were analyzed as PET parameters for 50 patients with primary lesions and 104 metastatic lymph nodes. SUVmax, which is widely used, shows only the most active point in a tumor and there is a limit in representing tumor heterogeneity or size. TLR, which supplemented the tumor’s SUV value with reference to liver activity, tried to overcome the heterogeneity of the other three PET-CT scanners in our study. Complementing this, MTV, which is a volume-based parameter, represents an area with a value above a certain SUV, and TLG represents tumor burden by multiplying MTV by mean SUV.

The volume-based parameters of the primary tumors were analyzed as prognosticators of survival and recurrence. Alluri et al. [24] reported that primary tumor MTV was a significant prognostic marker for event-free survival in multivariate analysis in stage III-IV HPV-positive oropharyngeal cancer patients treated with local or systemic treatment. The aim of the current study was to suggest the meaning of PET parameters in resectable oropharyngeal cancer patients with relatively non-bulky regional metastasis. The results showed that volume-based parameters of the primary tumor, such as MTV and TLG, were associated with OS and RFS. The volume-based parameters of the metastatic nodes did not show significant differences in OS or RFS, unlike those of the primary lesions. However, our study should be understood taking into account the inherent biases of the retrospective study design. Because of the limitation of small numbers of patients, metastatic lymph nodes also showed differences in survival in the survival curve, which need to be verified by further large-scale studies.

This study interestingly examined the association between surgical pathology and PET parameters. Previous research has been conducted on the accuracy of MRI in assessing DOI in oral and oropharyngeal cancers [25]. The studies showed that MRI was not clearly associated with the invasion depth of tonsillar cancer. However, in the current study, the volume-based parameters of PET and the DOI of oropharyngeal cancer, including tonsils, were correlated. Among them, TLG was a strong factor affecting the DOI of primary tumors. This demonstrated that higher primary tumor TLG meant a deeper depth of invasion and suggests that tumor invasion depth could be represented by volumetric functional imaging rather than by anatomic imaging such as CT or MRI alone in oropharyngeal cancer.

In addition, there are few studies of radiologic factors related to ENE, which are important factors in the prognosis of oropharyngeal cancer. A recent study divided the risk groups based on nodal MTV and ENE in laryngeal and hypopharyngeal cancers to explain the significant differences in survival analysis. Fujii et al. [26] analyzed the nodal MTV in PET-CT as a factor influencing the OS of patients with stage III/IV laryngeal or hypopharyngeal cancer who were treated with surgery, which showed stronger prognostic power than ENE. In the current study, additionally, the metastatic lymph nodes of oropharyngeal cancer were matched with the surgical pathologic findings, and the results showed that N-MTV was significantly associated with ENE in oropharyngeal cancer.

Recently, several studies on the de-intensification of treatment for HPV-positive oropharyngeal cancer have been actively conducted [27–29]. Previous studies analyzed the prognostic value of volumetric PET parameters associated with event-free survival in HPV-positive oropharyngeal cancer patients [18, 24, 30]. Chotchutipan et al. evaluated volumetric PET parameters in low-risk HPV-positive OPC patients treated with definitive chemoradiation, and analyzed the data to be prognostic of locoregional failure-free survival. The clinical stage was found to be a potent factor affecting distant metastasis-free survival or OS [30]. However, there has been no study to determine whether PET parameters associated with HPV positivity in oropharyngeal cancer. In the current study, there was no relationship between PET parameters and HPV infection, suggesting the limitation of PET as a useful tool for changes or de-intensification of treatment according to HPV status.

## Conclusion

In conclusion, volumetric parameters in PET were associated with survival, recurrence, invasion depth, and extranodal extension in oropharyngeal cancer. More evidence is needed before these findings are applied to the postoperative adjuvant treatment strategy for resected OPC patients. Further long-term follow-up assessments and prospective studies with large number of patients will be indicated in the near future.

## Data Availability

The dataset of the current study was available from the corresponding author on reasonable request.

## Abbreviations

PET: Positron-emission tomography
^18^F-FDG: ^18^F-fluorodeoxyglucose
CT: Computed tomography
SUV: Standardized uptake values
ENE: Extranodal extension
LN: Lymph node
DOI: Depth of invasion
HPV: Human papillomavirus
ROI: Region-of-interest
SUVmax: Maximum SUV
MTV: Metabolic tumor volume
TLG: Total lesion glycolysis
TLR: Ratio of the tumor-to-liver SUV
VOI: Volume of interest
ROC: Receiver operating characteristic
OS: Overall survival
RFS: Recurrence-free survival
T: Primary tumor
N: Metastatic node
tN: Total metastatic nodes
AUC: Area under the curve
CI: Confidence interval
